# GENDER DIFFERENCES IN COVID-19’S EFFECTS ON LONELINESS, ISOLATION, AND COGNITIVE HEALTH AMONG TAIWANESE RETIREES

**DOI:** 10.1093/geroni/igae098.3231

**Published:** 2024-12-31

**Authors:** Wan-Chen Hsu, Nuan-Ching Huang, Susan C Hu

**Affiliations:** National Cheng Kung University, Tainan, Tainan, Taiwan (Republic of China); National Cheng Kung University, Tainan, Tainan, Taiwan (Republic of China); National Cheng Kung University, Tainan, Tainan, Taiwan (Republic of China)

## Abstract

**Objectives:**

This study investigates the impacts of lock-down policies during the COVID-19 pandemic on the loneliness, social isolation, and cognitive function of retirees in Taiwan, focusing on the differences between men and women. Method: We conducted a two-stage stratified sampling survey to collect 1,115 valid retirees aged 50-74 in Taiwan in early 2023. A mixed-methods approach, combining focus groups and quantitative analysis, was employed. Loneliness was evaluated using the UCLA Loneliness Scale, and social isolation was measured through interaction with family members and social participation. Cognitive function was assessed using the Saint Louis University Mental Status. The statistical analysis included network analysis and multinomial logistic regression.

**Results:**

This study revealed that approximately 30% of participants experienced loneliness during the COVID-19 pandemic, and about 20% of men and women reported less time allocated to volunteering and group activities. Although regression analysis showed no gender differences in loneliness scores and cognitive functions, network analysis found gender-specific relationships among related factors and sub-item of cognitive tests. For example, women’s feelings of loneliness negatively correlated with geometric judgment performance, while men showed a positive correlation between orientation test scores and volunteer involvement. Qualitative research further underscored the crucial role of community support in facilitating recovery from the pandemic, particularly for individuals who were less engaged in community activities.

**Discussion:**

This study highlights the need to explore gender-specific correlates of loneliness and isolation across different cognitive items and emphasizes strengthening community support for post-pandemic recovery.

